# Copper(I)-Catalyzed Click Chemistry as a Tool for the Functionalization of Nanomaterials and the Preparation of Electrochemical (Bio)Sensors

**DOI:** 10.3390/s19102379

**Published:** 2019-05-24

**Authors:** P. Yáñez-Sedeño, A. González-Cortés, S. Campuzano, J. M. Pingarrón

**Affiliations:** Department of Analytical Chemistry, Faculty of Chemistry, Complutense University of Madrid, 28040 Madrid, Spain; aracelig@ucm.es (A.G.-C.); pingarro@quim.ucm.es (J.M.P.)

**Keywords:** copper(I) catalyzed click chemistry, azide-alkyne cycloaddition, electrochemical (bio)sensors, nanomaterials

## Abstract

Proper functionalization of electrode surfaces and/or nanomaterials plays a crucial role in the preparation of electrochemical (bio)sensors and their resulting performance. In this context, copper(I)-catalyzed azide-alkyne cycloaddition (CuAAC) has been demonstrated to be a powerful strategy due to the high yields achieved, absence of by-products and moderate conditions required both in aqueous medium and under physiological conditions. This particular chemistry offers great potential to functionalize a wide variety of electrode surfaces, nanomaterials, metallophthalocyanines (MPcs) and polymers, thus providing electrochemical platforms with improved electrocatalytic ability and allowing the stable, reproducible and functional integration of a wide range of nanomaterials and/or different biomolecules (enzymes, antibodies, nucleic acids and peptides). Considering the rapid progress in the field, and the potential of this technology, this review paper outlines the unique features imparted by this particular reaction in the development of electrochemical sensors through the discussion of representative examples of the methods mainly reported over the last five years. Special attention has been paid to electrochemical (bio)sensors prepared using nanomaterials and applied to the determination of relevant analytes at different molecular levels. Current challenges and future directions in this field are also briefly pointed out.

## 1. Introduction

Different types of the so-called click chemistry comprise cycloadditions of unsaturated species (1,3-dipolar cycloaddition reactions and Diels-Alder transformations), nucleophilic substitutions (ring-opening reactions of strained heterocyclic electrophiles), reactions involving non-aldol carbonyl group and addition reactions to carbon-carbon multiple bonds [1]. Among them, probably the most representative cycloaddition is the one catalyzed by copper(I) between an azide group and an alkyne (CuAAC), for which the term “click chemistry” is usually employed [2]. This reaction was introduced by Sharpless et al. in 2001 [3]. Although initially it was designed as a tool for organic synthesis [4,5], currently, this process is offered as one of the most useful methods for building architectures and incorporating functionalities into different materials. The high interest in the application of this reaction derives from the high yields it achieves, absence of by-product generation, high selectivity and moderate number of required conditions both in aqueous medium and under physiological conditions. The methodology, involving azide and alkyne complementary functions, is compatible with a wide range of *p*Hs, solvents and temperatures. Click reaction is considered also to be an efficient strategy for the immobilization of biomolecules (enzymes, antibodies and nucleic acids) to different substrates while maintaining their biological activity [6]. In the absence of a catalyst, cycloaddition is, mostly, non-regioselective and quite slow. The CuAAC rate is 10^7^ higher than the non-catalyzed reaction and can take place at room temperature [7]. Furthermore, various methodologies can be employed for catalyst incorporation based on the direct addition of copper(I) salts (chloride, iodide, acetate) to the reaction environment, or on the in situ production by the reaction of copper(II) salts (sulfate or acetate) with hydroquinone or ascorbic acid as reducing agents [8,9]. Moreover, Cu(I) catalyst can also be electrochemically generated [10]. This latter methodology is known as electroclick chemistry and can be used for the selective functionalization of electrode platforms [11]. Devaraj et al. demonstrated in 2006 the electrochemical control of the coupling catalyst Cu(I) to form 1,2,3-triazole between organic azides and terminal acetylenes by applying a potential of −300 mV versus Ag/AgCl yielding cuprous ions from copper(II) complexes. This approach was postulated as an ideal method for the modification of multielectrode surfaces without risk of contamination of the adjacent electrodes [12]. Hein and Fokin proposed the use of a copper piece to produce the catalyst, thus reducing contamination but requiring a long reaction time [8].

The exploitation of click chemistry to develop electrochemical sensors was reviewed in 2015 by Cernat et al. [5]. However, since that date, a noticeable increase in the number of reported methods has been produced, mainly for the preparation of biosensors for clinical purposes, and many of them have involved the use of nanomaterials. The current state of the art in this field is extensively reviewed in this article, with particular attention to electrochemical sensors and biosensors developed since 2015. The main characteristics of the prepared (bio)electrode platforms and their analytical performance and application to relevant samples are critically discussed and compared with other (bio)sensing strategies.

## 2. Preparation of Electrochemical Platforms

Proper preparation of the electrode platform prior to the construction of an electrochemical (bio)sensor is an inescapable and crucial step to achieving a good performance. The resulting surface must not only provide selective, repeatable and sensitive electrochemical responses, but also be able to allow the stable immobilization of the (bio)recognition element. In this context, click chemistry has been used as a versatile tool for different electrode materials functionalization using a variety of methodologies [13]. The characteristics and application of the recent configurations reported are summarized in Table 1 [10,14,15,16,17,18,19,20,21,22,23,24,25,26,27,28,29,30,31,32,33,34,35,36,37,38,39,40]. In the following sections they are discussed according to the different functionalization strategies.

### 2.1. Electrografting and Click Chemistry

**Figure 1:**
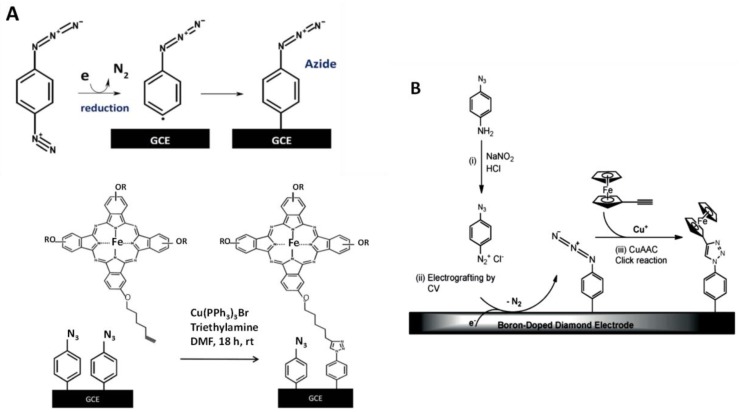
Functionalization of a GCE with azide groups by electrografting (top) and attachment of Fe(II) phtalocyanine on electrografted GCE via click chemistry (**A**); multistep functionalization of a BDDE by electrografting and attachment of ethynylferrocene via click chemistry (**B**). Reprinted from [28] (**A**) and [27] (**B**) with permission.

A frequent strategy for modifying electrode surfaces consists of electrografting and subsequent modification by CuAAC [41]. Aryldiazonium salts such as 4-azidobenzenediazonium have a particular interest in these strategies, since they may react with any compound with a terminal alkyne group under mild conditions with high specificity [42]. In a representative example, Nxele et al. [28] prepared glassy carbon electrodes (GCEs) grafted with terminal azide groups that were subsequently modified with an alkynyl tetra-substituted phthalocyanine through click reaction in the presence of Cu(I) (Figure 1A). The modified electrodes showed electrocatalytic ability towards the oxidation of hydrazine. A straightforward protocol for the covalent functionalization of boron-doped diamond electrodes (BDDEs) with ferrocene was based on 4-azidophenyl-diazonium chloride electrografting and further reaction with ethynylferrocene (Figure 1B). The resulting platform was used to incorporate single-stranded (ss)-DNA through reaction with an alkynyl-derivatized ss-DNA probe [27]. This method allowed multiplexed site-specific electrode functionalization for preparing multitarget biosensors on different materials [43].

### 2.2. Self-Assembled Thiol Monolayers (SAMs) 

Formation of alkanethiol SAMs on gold surfaces has been used as a prior step to the cycloaddition reaction. Collman et al. reported in 2004 an efficient surface functionalization using azide-terminated SAMs by CuAAC reaction [44]. More recently, terminal alkyne group-modified gold nanoparticles (AuNPs) were coupled with click chemistry in the presence of electrochemically generated Cu(I). The electro-assisted method allowed control of the working surface coverage [29]. AuNPs modified with 10-undecyn-1-thiol were immobilized on azide-modified GCEs prepared by grafting 4-azidoaniline diazonium salt and 1,3-dipolar cycloaddition through in situ Cu(I) generation [22]. Once the carbon substrate was modified with azide using diazonium chemistry, AuNPs were attached in the presence of Cu(II) and ascorbate. The AuNPs were responsible for the distortion of the 4-aminophenol monolayer, which allowed penetration of the hydrophobic ions and detection of the electronic exchange with the surface. This configuration allowed the detection of separate electrochemical signals for NO_2_^−^ and SO_3_^2−^.

**Figure 2:**
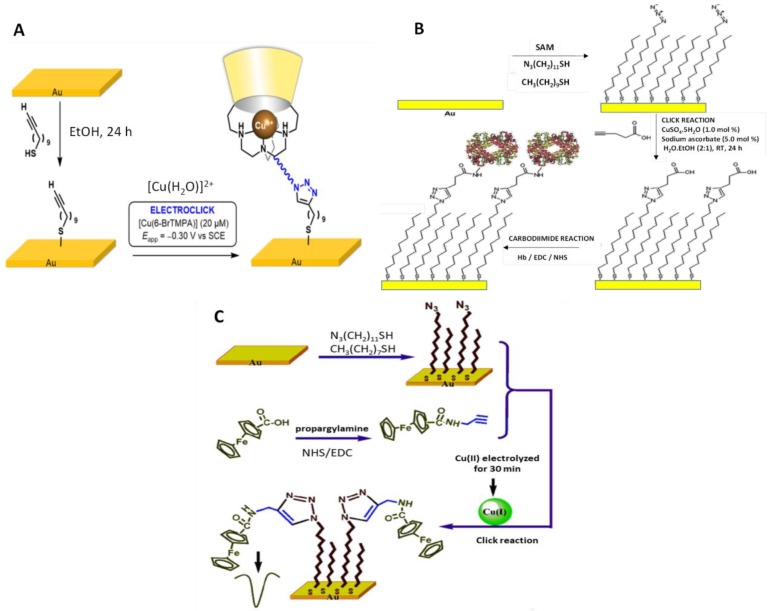
Strategy for electroclick grafting of [Cu(H_2_O)]^2+^ on gold electrodes modified with alkyne-terminated alkane thiols (**A**); Hb-functionalization of Au surface via click chemistry on mixed SAMs (**B**); fundamentals of copper(II) detection involving CuAAC electroclick (**C**). Reprinted from [33] (**A**), [14] (**B**) and [16] (**C**) with permission.

Electrochemically active SAMs have gained wider attention for their potential applications in nano-electromechanical systems (NEMS), charge/ion storage devices and biosensor fields. An illustrative example is the attachment of tetra(ethyleneglycol)-substituted phenyl-capped bithiophene with alkyne terminals (Ph2TPh-alkyne) on azide-terminated SAMs by Cu-catalyzed AAC reaction on a gold substrate. The resulting platform showed reversible electrochemical responses by cyclic voltammetry (CV) [45]. The copper calix [6] azacryptand funnel complex was immobilized onto an undec-10-yne-1-thiol-modified gold electrode by application of CuAAC electroclick methodology (Figure 2A). The open-shell cavitary property of the immobilized Cu complexes was exploited for the selective electrochemical detection of guest ligands by CV measurement of the corresponding Cu(II)/Cu(I) quasi-reversible response. This methodology allowed the selective detection of alkylamines at micromolar concentration [33]. Structurally well-defined organic SAMs were formed on Au surface from a mixture of azidoundecanethiol (N_3_C_11_H_22_SH) and 1-decanethiol using 4-pentynoic acid as a bifunctional linker to immobilized hemoglobin (Hb) via click and carbodiimide reactions (Figure 2B). The Hb-functionalized Au electrode exhibited direct electron transfer, showing a pair of well-defined and quasi-reversible peaks owing to the Fe(III)/Fe(II) redox couple at about −0.210 V versus saturated calomel electrode (SCE), as well as excellent electrocatalytic activity toward O_2_ and H_2_O_2_ [14]. Furthermore, an electrochemical sensor for Cu(II) was constructed based on the activity of cuprous ion as catalyst in the CuAAC reaction involving a similar SAM configuration with mixed N_3_C_11_H_22_SH and octylthiol assembled on the gold electrode surface (Figure 2C). Then, propargyl-functionalized ferrocene was covalently coupled on the electrode surface via click reaction. The resulting sensor exhibited a linear response to the logarithm of Cu(II) over the 1.0 × 10^−14^ to 1.0 × 10^−9^ mol L^−1^ range [16].

### 2.3. Carbon Nanomaterials

**Figure 3:**
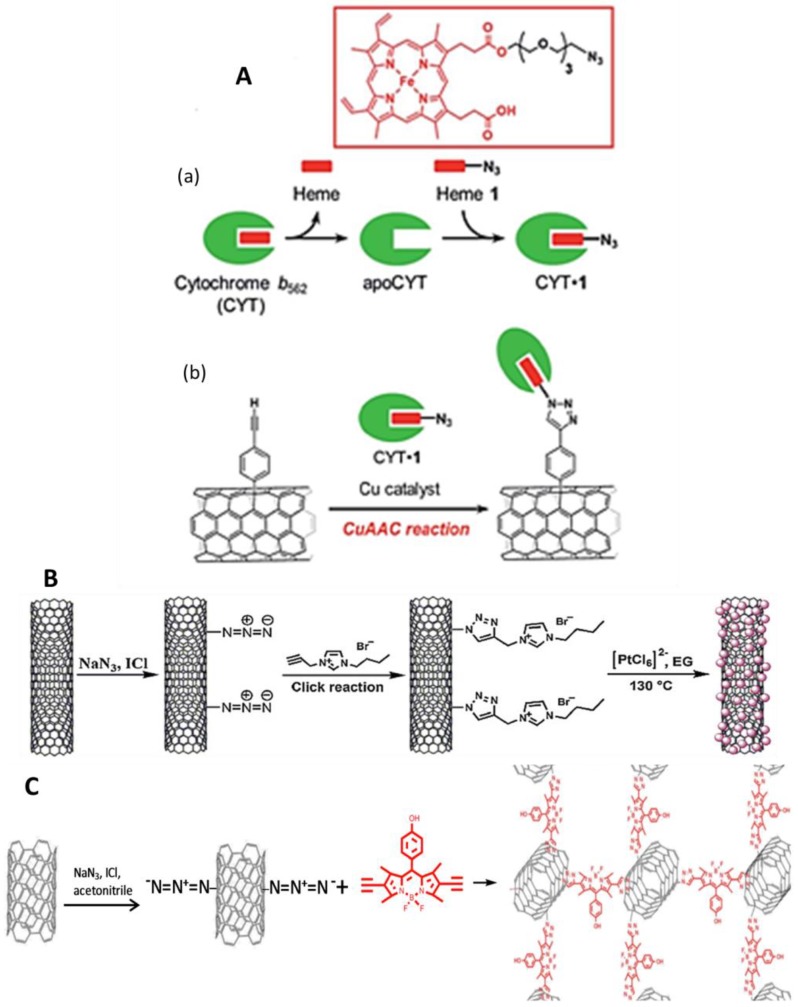
Preparation of CYT-1 (a) and CYT-1-SWCNTs (b) (**A**). Schematic diagram of modification of azido MWCNTs with IL and the preparation of MWCNTs–IL@PtNPs composite (**B**). Method for the preparation of 3D SWCNTs–BODIPY from azido-SWCNTs and double terminal ethynyl BODIPY (**C**). Reprinted from [47] (**A**), [26] (**B**), and [39] (**C**) with permission.

In the last years, the attractive electronic, chemical and mechanical properties of graphene and carbon nanotubes (CNTs) have been used in connection with click chemistry to enhance their properties. The adsorption capacity, the possibility to be functionalized and the ability to promote electron transfer reactions of many molecules make CNTs particularly attractive in developing electrochemical sensors and biosensors. Click chemistry has been demonstrated to be an ideal modular methodology for the incorporation of a wide variety of molecules on the surface of CNTs, and has been successfully applied to produce nanotube-based innovative materials. Campidelli [46] revised the application of CuAAC for CNT functionalization. In a more recent example, redox-active cytochrome b562 and a tethered azide group on the heme propionate side chain (cyt1) were covalently linked to an acetylene moiety introduced on the sidewall of single-wall carbon nanotubes (SWCNTs) by copper-catalyzed click chemistry (Figure 3A), forming a triazole ring with the heme active site directly linked to the carbon nanotubes [47]. CuAAC was also used in multi-walled carbon nanotubes (MWCNTs) functionalization with β-cyclodextrins (β-CDs) [48]. MWCNTs functionalized with the ionic liquid (IL) 1-propargyl-3-butylimidazoliumbromide via click chemistry (Figure 3B) were also prepared, and platinum nanoparticles (PtNPs) were loaded using ethylene glycol as a reducing agent. A GCE was coated with the MWCNTs-IL@PtNPs mixture and a molecularly imprinted polymer was developed by free radical polymerization using 4-vinylpyridine as unctional monomer and tartrazine as the template. The resulting molecularly imprinted polymer (MIP)–MWNTs-IL@PtNPs/GCE showed good analytical performance for the electrochemical determination of the food dye. The peak current was linear to tartrazine concentration in the 0.03–5.0 μmol L^−1^ and 5.0–20 μmol L^−1^ ranges with a detection limit (LOD) of 8 nmol L^−1^. The method was applied with good results for the analysis of commercial orange drinks and orange powder [26].

Borondipyrromethenes or BODIPYs (4,4-difluoro-4-bora-3a, 4a-diaza-s-indacenes) are of increasing interest due to their optical and electronic properties. A variety of derivatives have been synthesized, some of which, combined with carbon nanomaterials or conductive polymers, have been used for the construction of electrochemical sensors. As an example, a 3D SWCNTs-BODIPY hybrid material was synthesized via click reaction of a BODIPY-bearing double-terminal ethynyl groups with azido-containing SWCNTs (Figure 3C). The resulting SWCNTs-BODIPY/GCEs were applied to the determination of the pesticide eserine, a cholinesterase inhibitor, in aqueous solutions and orange juices by square wave voltammetry (SWV). Interestingly, the electrochemical reaction of eserine was found to be selectively electrocatalyzed by BODIPY derivative in the presence of many other pesticides. The peak current increased linearly with eserine concentrations in the 0.25–2.25 μM range with an LOD of 160 nmol L^−1^ [39]. The same group reported the synthesis and electrochemical properties of other hybrids prepared from azido-SWCNTs covalently functionalized with different ethynyl-BODIPY derivatives by CuAAC, as well as their responses toward guanine and adenine. The hybrid provided a better response, and using differential pulse voltammetry (DPV) it yielded LODs of 1.07 and 18.8 μmol L^−1^ for guanine and adenine, respectively. The resulting SWCNTs-BODIPY/GCE was used for quantification of a double-stranded (ds)-DNA sample from calf thymus [38].

In the case of graphene, click chemistry is also an important strategy for the covalent linking of different compounds on a substrate via complementary azide or alkyne groups. Cernat et al. [49] modified GO with azide groups, generating a new material with active sites available for click chemistry reactions. The azide group was inserted into the graphene oxide backbone by chemical functionalization. The successful synthesis of the graphene-azide platform was validated by electrochemical methods after clicking the electroactive model molecule ethynylferrocene. In an interesting paper, Shen et al. [50] immobilized graphene on an azide group-modified gold surface via π–π interaction and click reaction. Alkyne-functionalized pyrene was used as a molecular “anchor” to adsorb on graphene forming π–π staking structure without affecting the graphene sheets. Then, the alkyne terminal group was reacted with azide via CuAAC to form the graphene-gold conjugate.

### 2.4. Phtalocyanines

Metallophthalocyanines (MPcs) exhibit good electrocatalytic activity for many analytes such as nitrite, dopamine, hydrazine and amitrole due to the accessibility of a range of oxidation states on the ring and some central metals such as Co, Ni, Fe and Mn [28]. Drawbacks such as the lack of selectivity of chemically modified electrodes prepared with MPcs can be avoided or minimized by means of click chemistry, since this provides stable monolayer thin films onto electrode surfaces. Tetrakis (5-hexyn-oxy) Fe(II) phthalocyanine was synthesized to perform a click reaction between the terminal alkyne groups and an azide group on a GCE surface. The azide group was formed through electrografting using 4-azidobenzene diazonium tetrafluoroborate by electrochemical reduction. The Cu(I)-catalyzed alkyne-azide Huisgen cycloaddition reaction was used to react the terminal alkyne groups on the phthalocyanine with the azide groups on the GCE surface. The resultant electrode demonstrated good electrocatalytic ability towards hydrazine oxidation and provided an LOD of 1.09 μM [28].

**Figure 4:**
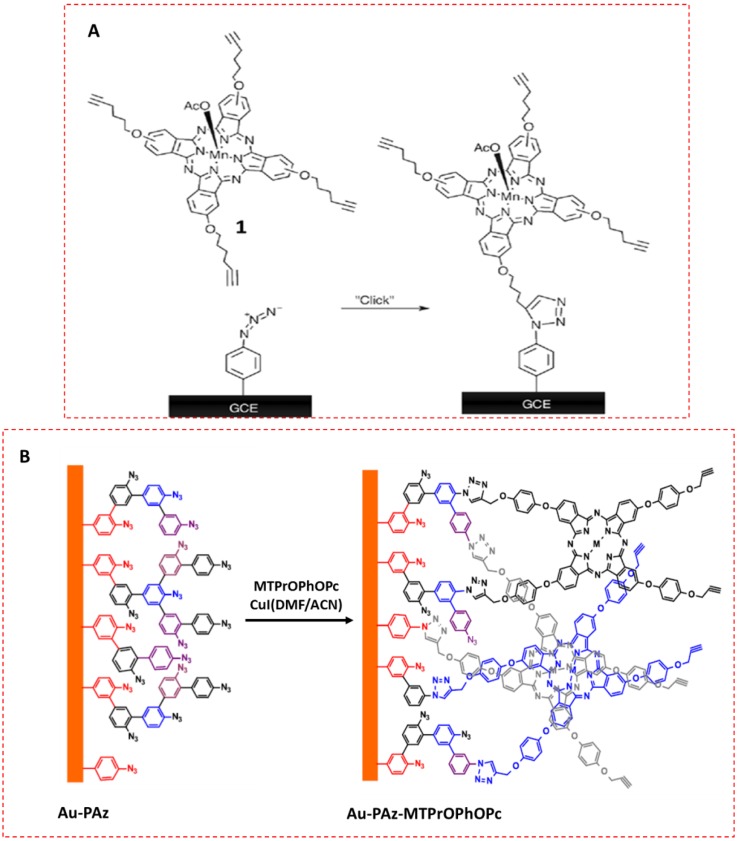
Clicking of (OAc)Mn tetrakis(5-hexyn-oxy) phtalocyanine to a grafted GCE (OAc: acetate) (**A**). Scheme of electrochemical grafting and click reaction of MTPrOPhOPcs onto grafted AuE (**B**). Reprinted from [32] (**A**) and [34] (**B**).

An interesting strategy proposed by Ipek et al. involved the immobilization of a terminal-alkynyl cobalt phthalocyanine (CoPc-TA) on a GCE modified with electropolymerized 4-azidoaniline (N_3_-PANI). The resulting electrode, CoPc-TA/N_3_-PANI/GCE, showed electrocatalyzed responses towards the detection of some pesticides such as eserine [10]. SWV provided an LOD of 0.175 μmol L^−1^ for this pesticide. More recently, clicking of manganese tetrahexynyl phthalocyanine by CuAAC was also performed on a GCE previously grafted with a diazonium salt-containing azide group (Figure 4A). In this case, the LOD value achieved for hydrazine, 15.4 *p*mol L^−1^, constituted an important improvement compared to that obtained with other reported sensors for this analyte [32], such as, for instance, a grafted GCE by 1, 3-dipolar cycloaddition reaction modified with a cobalt tetrakis 4-((4-ethynylbenzyl) oxy) phthalocyanine, which gave an LOD of 3.28 μmol L^−1^ [35].

An electrochemical sensor for the detection of mercury(II), lead(II), copper(II) and cadmium(II) ions was reported using a synthesized low-symmetry alkyne-terminated cobalt phthalocyanine (CoPc) derivative. Differential pulse stripping voltammetry (DPSV) was employed for the simultaneous determination of trace levels of the above-mentioned metal ions using modified GCE via click chemistry. The anodic peak current was proportional to the concentrations of metal ions over a wide range up to 0.1 mmol L^−1^ without interferences from the different metal ions [37]. Terminal alkynyl-substituted manganese phthalocyanine (TA-MnPc) was bound to 4-azido polyaniline (PANI-N_3_) on an indium thin oxide coated glass (ITO) electrode by using click electrochemistry to prepare a PANI-N_3_/TA-MnPc hybrid that was used for the SWV detection of fenitrothion pesticide with an LOD value of 49 nmol L^−1^ [36].

Tetra-(4-propargyloxy)phenoxy phthalocyanines (MTPrOPhOPc) were covalently immobilized as thin films onto gold surfaces via click reaction (Figure 4B). Gold electrode surfaces were pre-functionalized with phenylazide (Au-PAz) thin film using in situ diazonium generation followed by electrografting. CuAAC reaction was used to covalently immobilize MTPrOPhOPcs onto the gold electrode to form Au-PAz-MTPrOPhOPc. The electrocatalytic and electroanalytical properties of the modified electrodes were tested towards the detection of hydrogen peroxide, achieving an LOD in the μM range. In addition, Au-PAz-MTPrOPhOPc exhibited good reproducibility and stability in various electrolyte conditions [34].

Quantum dots (QDs) are semiconductor nanocrystals with high electrocatalytic ability that can be used alone or combined with other molecules for the electrocatalysis of different compounds [51]. Azide-functionalized CdSe/ZnS QDs conjugated with tetrakis (5-hexyn-oxy) Fe(II) phthalocyanine (FePc) were used for the electrocatalytic detection of paraquat (1, 1-dimethy l-4, 4-bipyridinium dichloride), a toxic pesticide highly resistant to biodegradation. An increase in the CV peak currents was observed at the QDs-FePc/GCE modified electrode, where the Fe(II)/Fe(I) redox pair of the phthalocyanine catalyzed the reduction of paraquat. An improved LOD of 5.9 nmol L^−1^ compared to other sensors for this analyte was reported [31].

### 2.5. Polymers

Ferrocene-containing polymers (FcPs) have attracted much interest in the last decade due to their unique features and wide applications. Here, click chemistry has resolved the great synthetic challenge to introduce the functional group in the polymer network. As an example, Scavetta et al. [23] reported the synthesis of Fc-functionalized poly (3,4-ethylenedioxythiophene) (PEDOT-Fc) via CuAAC. The resulting conducting polymer exhibited a relatively fast electron transfer rate. ITO electrodes were modified through electrodeposition of PEDOT-N_3_ followed by CuAAC with ethynylferrocene. The Fc-decorated electrode was used as an amperometric sensor for dopamine with an LOD of 1 μmol L^−1^. In another work, Fc-functional thiophene (Thi-Fc) and Fc side-functional styrene copolymer (P3) were used as chemical probes for the electrochemical sensing of phosphate anions. These Fc-functional molecules were built through a 1,3-dipolar cycloaddition reaction between azide-functional groups of their precursors and ethynylferrocene. CV experiments showed that both Thi-Fc and P3 have diffusion-controlled redox behavior and the Fe^2+^/Fe^3+^ redox couple undergoes a quasi-reversible process. The modified electrodes responded selectively to the addition of H_2_PO_4_^−^ and HP_2_O_7_^3−^ anions in the form of their n-Bu_4_N^+^ salts by shifting their redox potentials cathodically [21].

Composites of graphene and poly (3, 4-ethylenedioxythiophene)-polystyrene sulfonate (PEDOT:PSS) were obtained by chemical modification via click chemistry under mild conditions with the goal to improve the electrical conductivity of polymer. Alkyne functionalized-graphene and PEDOT:PSS-bearing azide moieties reacted via click chemistry at room temperature for 48 h using copper sulfate as catalyst [52]. Molecularly imprinted polymer thin films on Au electrodes were prepared by the covalent attachment of monomer molecules to SAM-modified electrodes using click chemistry. Wang and Shannon [17] clicked propargyl acrylate onto an azidoundecanethiol/decane-thiol mixed SAM and, after applying UV light (365 nm) in the presence of *N*,*N*-methylenebis (acrylamide) and azobisisobutyronitrile as the radical initiator, polymerization was performed directly on the electrode surface in the presence of hydroquinone (HQ) as the electroactive template molecule. The chronoamperometric detection of HQ with the clicked-on MIP sensor provided an LOD of 1.21 ± 0.56 μmol L^−1^.

**Figure 5:**
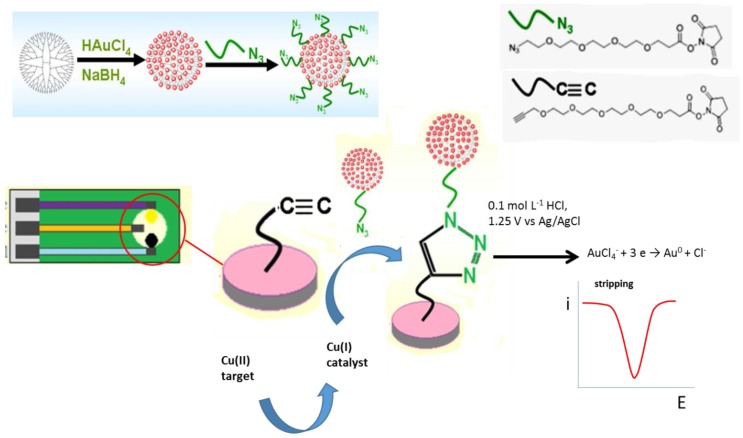
Schematic illustration of the anodic stripping voltammetric detection of Cu^2+^ ions by coupling with a Cu(I)-catalyzed azide alkyne click reaction and AuNP-PAMAM signal amplification. Reproduced from [30] with permission.

Dendrimers are branched polymers with high-density surface functional groups that have been applied in the construction of electrochemical sensors, usually by combining with nanomaterials. Figure 5 displays a platform for the detection of Cu(II) developed by coupling click chemistry with nanogold-functionalized poly(amidoamine) (PAMAM) dendrimer (AuNPs-PAMAM). The catalyst Cu(I) was synthesized by reduction of Cu(II) with ascorbate, and the azide-modified AuNPs-PAMAM covalently conjugated via CuAAC reaction to an acetylene-modified screen-printed carbon electrode (SPCE). The detection method involved treatment of the gold conjugate by oxidation in acidic medium followed by stripping voltammetry of the oxidized AuCl_4_^−^ ions. The resulting response relied directly on copper ion concentration in the sample. The method achieved an LOD as low as 2.8 *p*mol L^−1^ Cu(II) [30].

## 3. Electrochemical Biosensors Prepared using CuAAC

CuAAC has drawn increasing attention in electrochemical biosensing as a route for effective surface modification, bioconjugation and signal transformation or amplification. In this field, mild conditions of reaction, high selectivity and yields are the most prominent advantages of click chemistry that allow attaching proteins, enzymes, viruses, bacteria and cells to different materials. Table 2 summarizes recent examples of biosensors reported in the literature [53,54,55,56,57,58,59,60,61,62,63,64,65,66,67,68,69,70,71,72,73,74,75,76,77,78,79].

### 3.1. Enzyme Biosensors

The application of click chemistry for the design of enzyme biosensors relies on the controlled immobilization of catalytic compounds on a specific center onto the detection platform. Although both ethynyl and azide groups, the partners involved in the CuAAC reaction, have a high energy level, they are inert to biomolecules, and ensure their immobilization at specific sites on solid surfaces, thus contributing to the development of third-generation biosensors based on direct electronic transfer (DET) [5]. A common design of electrode surface modification with enzymatic compounds involves the detection of hydrogen peroxide. Due to its stability and easy functionalization with both azide and alkyne groups, horseradish peroxidase (HRP) is the most-used enzyme. The tagged enzyme immobilization can be performed at room temperature on various conductive materials while maintaining its bioactivity [80]. The electrochemical detection can be performed indirectly through redox probes, or directly by means of the electron transfer (ET) between the enzyme active center and the electrode surface

**Figure 6:**
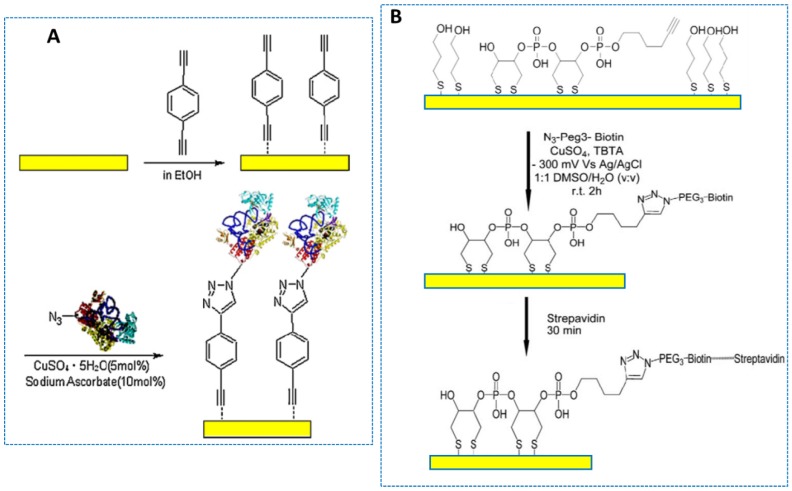
Preparation of a HRP-DEB biosensor (**A**). Functionalization of gold surface with bis(DTPA)-hexynyl and mercaptopropanol; biotin grafting by click reaction followed by streptavidin binding (**B**). Reproduced from [53] (**A**) and [59] (**B**) with permission.

ET in redox proteins is key to many biological processes and of great interest in the preparation of electrochemical biosensors. However, since the redox protein must be immobilized on the electrode, direct ET is not easy to realize. In this context, click chemistry has been shown to open a way to enhance the direct ET between a redox protein and the underlying electrode. An illustrative example is the simple and versatile approach for covalent immobilization of HRP onto a gold electrode via self-assembled and click chemistry. Figure 6A shows that the alkynyl-terminated monolayers of 1, 4-dialkynyl benzene (DEB) were self-assembled, and azido-HRP was covalently immobilized onto the formed monolayers by click reaction. The enzyme biosensor exhibited electrocatalytic reduction for H_2_O_2_ through the linear range from 5.0 to 700 μmol L^−1^ [53]. In a more recent configuration, an azide-PEG3-biotin derivative was grafted onto a gold electrode modified with a mixed hexynyl-terminated DTPA (dithiol phosphoramidite) and a mercaptopropanol monolayer (Figure 6B). The azide-PEG3-biotin was grafted in the presence of Cu(II), which was electrochemically reduced to Cu(I) at −0.3 V versus Ag/AgCl. The prepared platform was used for the immobilization of streptavidin and the coupling of biotinylated human serum albumin (HAS). Protein detection by electrochemical impedance spectroscopy (EIS) provided a detectable concentration of 10 *p*g mL^−1^ HAS [59].

### 3.2. DNA Biosensors

Electrochemical DNA biosensors have gained popularity with respect to other methods for DNA analysis due to their rapidity, high sensitivity and cost-effectiveness [72]. Among others, methods involving hairpin-like oligonucleotides are being extensively used due to inherent simplicity and specificity. In this context, CuAAC has found broad application for surface functionalization and the immobilization or conjugation of oligonucleotides, taking advantage of mild reaction conditions and excellent chemoselectivity and orthogonality to provide a wide variety of functional groups. Moreover, with the aim of avoiding the synthesis of Cu(I) catalyst by chemical reduction of Cu(II), which could cause the oxidative cleavage of DNA by the highly reactive hydroxyl radicals generated, electrochemical reduction in the absence of ascorbic acid is usually utilized.

**Figure 7:**
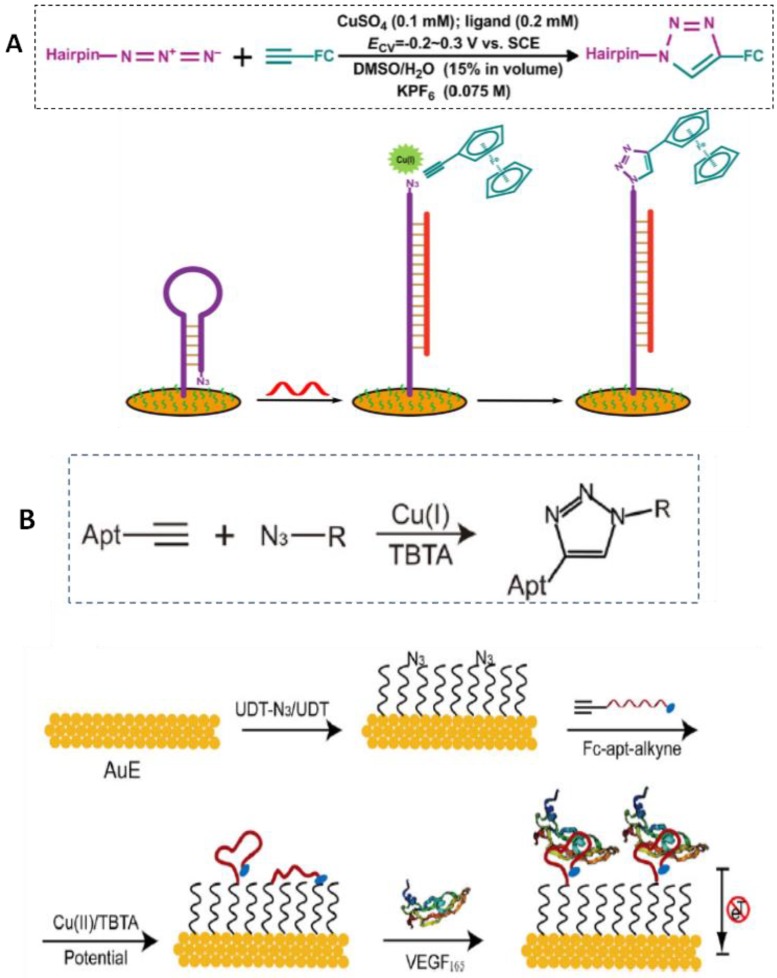
Schematic illustration of electrochemical DNA biosensors prepared onto gold electrodes by direct immobilization of dually thiol- and azide-labeled probes, followed by hybridization and electroclick reaction (inset) with ethynilferrocene (**A**). Fabrication of a mixed monolayer of 11-azido-1-undecanethiol (N_3_UDT) and UDT, followed by electroclick (inset) with Fc and the alkyne-modified probe, and target binding (**B**). Reprinted from [65] (**A**) and [73] (**B**) with permission.

One of the DNA biosensors proposed by Hu et al. can serve as a generic example illustrating a scheme of the signal-on detection of sequence-specific DNA using potential-assisted CuAAC for the labeling of hairpin-like oligonucleotide with an electroactive probe [72]. Figure 7A shows that the hairpins, dually labeled with terminal thiol and azide moieties, were self-assembled on a gold electrode and served as the capture probes for the specific recognition of target DNA (not related to any particular analyte). Upon hybridization, the unfolded surface-confined hairpins liberated the azide-containing terminals away from the electrode surface. Subsequently, the unfolded hairpins were labeled with ethynylferrocene (EFC) via CV electroclick, and the quantitatively labeled EFC was measured by DPV for the signal-on detection of the sequence-specific DNA. The biosensor exhibited a good linear response over the range from 1 to 1000 *p*mol L^−1^ with an LOD of 0.62 *p*mol L^−1^, and was successfully applied to the analysis of serum samples [65]. A DNA biosensor for the determination of thrombin, a serine protease with an important role in the coagulation cascade, was also reported by the same group using this strategy [76].

The preparation of DNA biosensors by CuAAC reaction onto electrode surfaces previously modified with alkanethiol monolayers has been suggested by some authors as a better alternative to the use of directly adsorbed thiolated probes, because it leads to well-ordered and stable receptor monolayers, avoiding pinhole formations surrounding the oligonucleotide. Usually, these configurations are prepared by the initial treatment of the gold surface with a mixture of azide terminal alkanethiol and blocking alkanethiol, followed by the addition of the alkynylated sequence, which is conveniently linked by the click reaction catalyzed by Cu(I) ions. A covalent link between the mixed azide–alkanethiol monolayer and the DNA molecules is established without disturbing the structural integrity of the monolayer. An example is illustrated in Figure 7B. Feng et al. [73] used this strategy in the development of a biosensor for the determination of vascular endothelial growth factor 165 (VEGF165), a protein playing a key role in the process of tumor angiogenesis and neuron diseases. In this case, Cu(II) ions were reduced by application of a potential of −0.5 V versus Ag/AgCl directly on the modified surface in the presence of a stabilizing ligand, tris[(1-benzyl-1*H*-1,2,3-triazol-4-yl) methyl] amine (TBTA). This method allowed an easy control of the surface aptamer density, with a high coverage and stability, providing an LOD of 6.2 nmol L^−1^.

**Figure 8:**
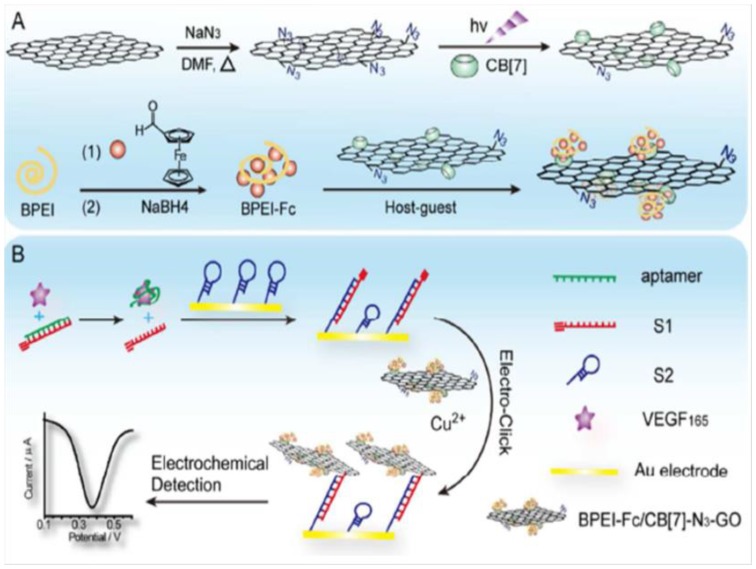
Preparation of BPEI-Fc-CB[7]-N_3_-GO composite (**A**) and schematic illustration of the electroclick biosensing platform constructed for the determination of VEGF165 (**B**). Reprinted from [75].

A more sensitive electrochemical DNA biosensor for VEGF165 was also prepared using electroclick chemistry onto a surface composed of cucurbit[7]uril (CB[7]) and azide co-functionalized graphene oxide (N_3_-GO). As Figure 8 A shows, CB[7] was immobilized onto N_3_-GO through photocrosslinking with a part of the azide groups, whereas the remaining groups were attached to the alkyne functionalized DNA. Furthermore, CB[7]-N_3_-GO was functionalized with Fc-grafted branched-ethylene imine polymer (BPEI-Fc) signal tags. Separately (Figure 8B), a VEGF165 aptamer probe was hybridized with the alkyne-functionalized S1 probe in the sample solution. In the presence of the target protein, the S1 probe was displaced and therefore could hybridize with a hairpin DNA probe (S2) immobilized on a gold electrode that unwound the hairpin structure. Then, electroreduction of Cu(II) at the electrode provided Cu(I) catalyzed the intramolecular organic azide–alkyne cyclization and the linking of BPEI-Fc/CB[7]-N_3_-GO composites, resulting in an amplified electrochemical detection signal. The biosensor provided a dynamic range from 10 fg mL^−1^ to 1 ng mL^−1^ and an LOD of 8 fg mL^−1^ VEFF165 [75].

Electrochemical DNA sensors involving the use of conducting polymers (CPs) have been prepared to detect DNA hybridization events in a label-free approach, taking advantage of the high sensitivity of CPs’ electronic structure that permit environmental changes to occur at the polymer surface. Electropolymerized CPs with functional groups for coupling with the DNA probe have been used and, among the coupling reactions, the chemoselective CuAAC of azides with terminal alkynes has shown to be a useful strategy. An illustrative example is the label-free electrochemical DNA sensor using azidomethyl-derivatized PEDOT-modified electrodes for the detection of a sequence correlating with the hepatitis C virus (HCV), proposed by Galán et al. [62]. An acetylene-terminated oligonucleotide probe, complementary to an HCV target sequence, was immobilized onto an azido-PEDOT polymer by covalent binding using click chemistry. DNA hybridization was detected by DPV measurements of the changes in the electrochemical properties of the polymer triggered by the recognition event. An LOD of 0.13 nmol L^−1^ was achieved.

**Figure 9:**
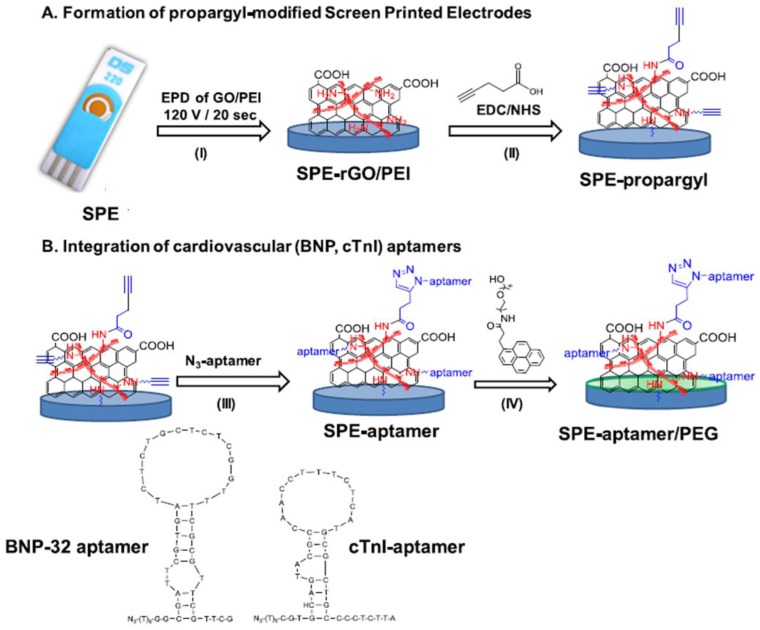
Steps involved in the construction of an electrochemical aptasensor for BNP and cTnI cardiac biomarkers. Preparation of gold SPE-rGO/polyethyleneimine (PEI) by electrophoretic deposition (EPD) of rGO/PEI onto gold screen-printed electrodes (I) and covalent binding of propargylacetic acid and PEI (II) (**A**). Integration of N_3_-functionalized aptamers by click chemistry (III), and blocking with pyrene-polyethylene glycol (PEG) (green layer) (**B**). Reproduced from [78] with permission.

Cu(I) click chemistry has also been utilized for the preparation of multianalyte DNA-sensing platforms. For example, Levrie et al. [43] combined the electro-addressability of diazonium salts electrografting with the chemoselectivity of click chemistry for the site-specific immobilization of two different ssDNA probes side by side on a single chip. Two different aryldiazonium salts, 4-ethynylaniline and 4-azidoaniline, were adjacently grafted on an electrode array chip for further site-specificity, coupling alkyne- and azide-modified ssDNA on the functionalized electrodes. Another strategy is the preparation of a multiplexed biosensor for cardiac biomarkers involving aptamer-based electrodes. Figure 9 shows that screen-printed gold electrodes were modified with rGO/polyethyleneimine (PEI), followed by covalent grafting of propargylacetic acid to integrate propargyl groups onto the electrode. Azide-terminated aptamers for brain natriuretic peptide (BNP) and cardiac troponin I (cTnI) were then immobilized by CuAAC. The developed biosensor showed a linear response from 1 *p*g mL^−1^ to 1 μg mL^−1^ for BNP, and from 1 *p*g mL^−1^ to 10 ng mL^−1^ for cTnI [78].

**Figure 10:**
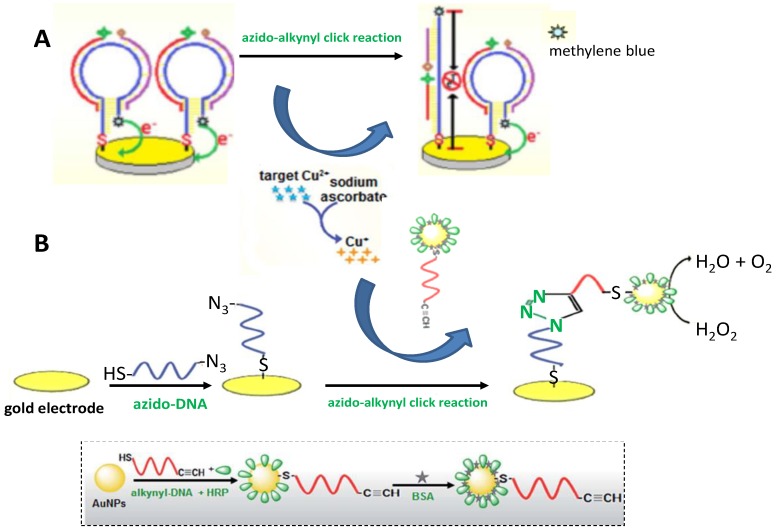
Schematic illustrations of the sensing strategies reported for copper ion detection by target-induced click conjugation of two oligonucleotides labeled with azides and alkynes using methylene blue-functionalized hairpin DNA as the template (**A**) or HRP as indicator and AuNPs as enhancer (**B**). Inset: preparation of alkynyl-DNA/HRP/BSA/AuNPs. Reprinted from [64] (**A**) and [74] (**B**) with permission.

Using hairpin DNA-dependent click conjugation of oligonucleotides, electrochemical sensing platforms were designed for the sensitive detection of copper(II). The method reported by Tang et al. [64] involved two short oligonucleotides conjugated by CuAAC and methylene blue (MB)-functionalized hairpin DNA as the template (Figure 10A). The analyte (Cu^2+^) reduced in situ by sodium ascorbate to Cu^+^ catalyzed the click conjugation between two single-stranded oligonucleotides labeled with 5′-alkyne or 3′-azide groups. This provided a long-chain oligonucleotide inducing the opening of the hairpin DNA, which placed the MB tag far away from the electrode. The decrease in the MB current was specifically and directly proportional to Cu^2+^ concentration, allowing the detection of the ion at a concentration as low as 1.23 nmol L^−1^. More recently, the same group [74] reported the Cu(II) detection at the attomolar level using target-induced click conjugation of alkynyl-DNA/HRP-labeled AuNP and immobilized azido-DNA on the electrode (Figure 10B). Once the target Cu(II) ion was reduced to Cu(I) with ascorbic acid to catalyze the azide–alkyne click reaction, the carried HRP molecules could electrochemically reduce the substrate (H_2_O_2_), resulting in a large electrochemical current indirectly related to copper ion concentration. The as-prepared sensor exhibited a dynamic working range from 1.0 amol L^−1^ to 10 μmol L^−1^ with an LOD of 0.38 amol L^−1^.

**Figure 11:**
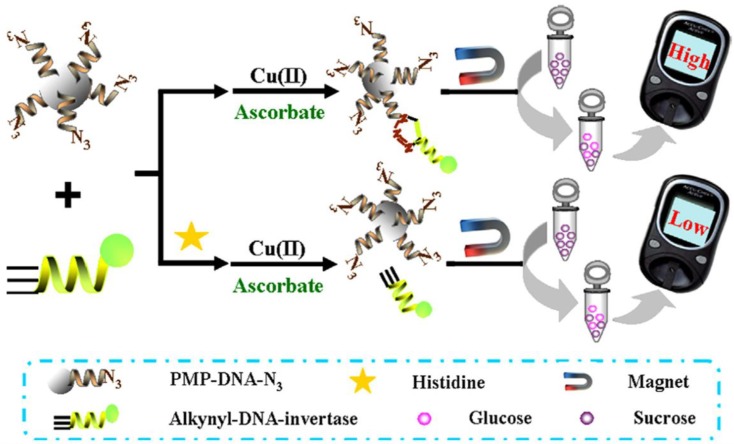
Schematic display of the portable chemical sensor for the determination of histidine, making use of click chemistry and PGM. Reproduced from [81] with permission.

Histidine is one of the natural amino acids playing an essential role in the growth and repair of tissues and in the mammalian central nervous system. An electrochemical biosensor for the determination of histidine involving click chemistry was prepared making use of conventional portable glucose meters (PGMs) (Figure 11). The method involved the invertase-labeled alkynyl-DNA immobilization on the surface of azide-DNA-modified paramagnetic particles. The Cu(II) reduction to Cu(I) was inhibited in the presence of histidine and Cu(II), and a decrease in the PGM signal was observed [81].

### 3.3. Immunosensors

**Figure 12:**
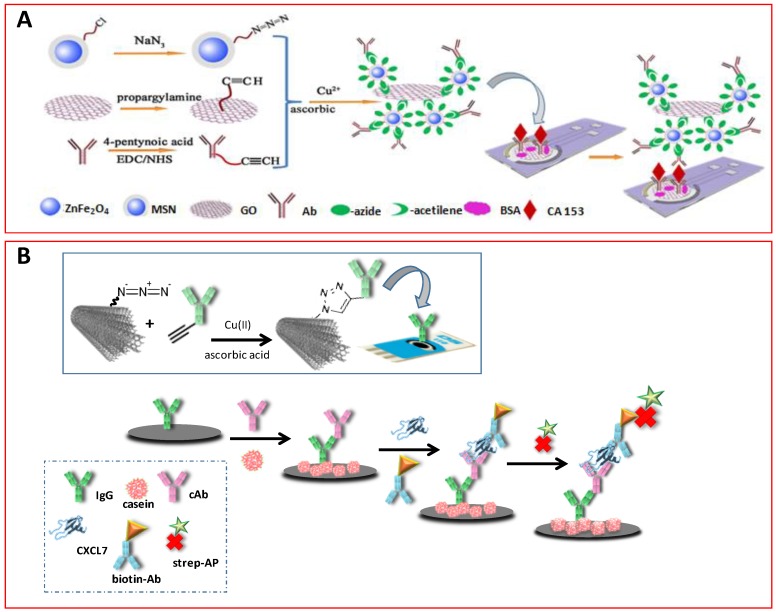
Schematic illustrations of the steps involved in the preparation of electrochemical immunosensors for CA 153 (**A**) and CXCL7 (**B**). See text for details. Reprinted from [60] (**A**) and [79] (**B**) with permission.

The opportunity to covalently couple biomolecules at specific sites through a fast and selective method and the resistance to side reactions makes click chemistry particularly attractive also in immunosensor construction. Various CuAAC applications reported in the literature show the usefulness of nanomaterials for the construction of electrochemical immunosensors both by employing them as electrode modifiers for immunoreagents binding by click chemistry, or in designing carrier tags for sensitive detection. An illustrative example involves the preparation of silica-coated ferroferric oxide (Fe_3_O_4_@SiO_2_) nanoparticles followed by functionalization with sodium azide, and their use in the construction of an immunosensor for the determination of microcystin-LR (MLR), the most toxic species of cyanotoxin, coupling alkyne-functionalized antibody and HRP by click reaction. After the sandwich immunoreaction, the determination of MLR was performed through HRP and Fe_3_O_4_ catalysis of the thionine (THI) oxidation in the presence of H_2_O_2_. A detection range between 0.01 and 200 μg mL^−1^ with an LOD of 0.004 μg mL^−1^ was reported [56]. The same group prepared a disposable electrochemical immunosensor for antigen 153 (CA 153, a breast cancer-related biomarker) determination, using CuAAC to synthesize graphene oxide (GO)-magnetic silica nanoparticle (MSN)-secondary antibody (Ab_2_) conjugates as detection labels (Figure 12A). A sandwich-type configuration was developed by covalent immobilization of capture antibodies onto GO/SPCEs. The signal measurement was carried out upon addition of H_2_O_2_ in the presence of THI as the redox mediator, profiting the peroxidase-like catalytic activity of MSNs toward the redox system. The response varied linearly, with a logarithm of CA 153 concentration from 10^−3^ to 200 U mL^−1^, and an LOD of 2.8 × 10^−4^ U mL^−1^ [60].

An electrochemical immunosensor for the determination of transforming growth factor β1 (TGF-β1), a multifunctional cytokine involved in various types of cancer and autoimmune diseases, was implemented using MWCNT-modified SPCEs. MWCNTs were functionalized by means of Cu(I)-catalyzed azide–alkyne cycloaddition as an efficient strategy for the covalent immobilization of the capture antibody. Alkyne-functionalized IgGs were synthesized to assemble IgG-alkyne–azide–MWCNT conjugates which were used as scaffolds for the immunosensor preparation. A sandwich-type configuration was developed with biotinylated anti-TGF labeled with poly-HRP-labeled streptavidin. The affinity reaction was monitored amperometrically at −0.20 V using the hydroquinone (HQ)/H_2_O_2_ system. The calibration plot for TGF-β1 exhibited a range of linearity, extending between 5 and 200 *p*g mL^−1^, which is suitable for the determination of the target cytokine in human serum. An LOD of 1.3 *p*g mL^−1^ was achieved, and the immunosensor demonstrated applicability to the analysis of spiked human serum samples at different concentration levels [69].

More recently, a similar strategy was applied for preparing the first electrochemical immunosensor for CXCL7 (chemokine (C-X-C motif) ligand 7, an autoimmune biomarker related to rheumatoid arthritis) determination (Figure 12B). After immobilization of the capture antibodies onto IgG-MWCNTs conjugates, the target antigen was sandwiched using biotinylated detector antibodies further conjugated with alkaline phosphatase (AP)-streptavidin conjugate. For the analytical readout, DPV was used in connection with 1-naphthylphosphate. Results presented demonstrated a linear calibration plot between 0.5 and 600 *p*g mL^−1^ CXCL7 and an LOD of 0.1 *p*g mL^−1^. This immunosensor also offered successful performance for the accurate determination of the endogenous levels of CXCL7 in serum samples from patients with rheumatoid arthritis [79].

### 3.4. Other Electrochemical Biosensors

Although peptides have interesting features as capture ligands, their use as probes in the preparation of electrochemical biosensors is relatively recent. Natural or synthetic polymers of amino acids with shorter lengths than those of proteins generally have better chemical and conformational stability, and specific sequences can provide high affinity to given analytes. Further advantages include high stability, easy modification, large chemical versatility and standard synthetic protocols, when needed [82]. Unfortunately, since peptides have small molecular mass and often lack a well-defined three-dimensional structure, they are not easily accessible when non-specifically immobilized on solid supports, and require a correct orientation to promote their interaction with a target.

A peptide-based biosensor for the label-free electrochemical detection of prostate-specific antigen (PSA) was constructed using a specific seven-amino-acid sequence (HSSKLQL). An electrode platform constituted of a GCE electrografted with the diazonium salts of two anilines was utilized to covalently immobilize the ethynyl-functionalized peptide probe through click coupling. A hydroxy naphtoquinone derivative was employed to electrochemically transduce the peptide-protein recognition event. In this configuration, two detection schemes were investigated based on the complexation or cleavage of the peptide by PSA. When the peptide was recognized by PSA, the SWV current from the redox quinone-derivative aniline decreased as a consequence of the electrode surface hindrance. On the contrary, upon cleavage by PSA of a biotinylated probe carrying streptavidin at its distal end, the biotin-streptavidin conjugate was liberated to solution, which freed the electrode surface and increased the electrochemical response. This latter approach allowed the detection of PSA over the 10^−12^ to 10^−6^ mol L^−1^ range [70].

Matsubara et al. [71] developed a peptide-based electrochemical biosensor for the detection of influenza virus (IFVs) using an alkyne-terminated boron-doped diamond (BDD) electrode. This approach, instead of the common sialyloligosaccharide receptor of influenza A and B viruses required during the early phase of infection, used a dimer of the sialic acid-mimic peptide as receptor with an azide lysine to be linked to the alkyne-terminated BDD electrode by CuAAC. EIS was utilized for the detection of seasonal H1N1 and H3N2 virus subtypes in the range of 400–8000 plaque-forming units (pfus) mL^−1^.

**Figure 13:**
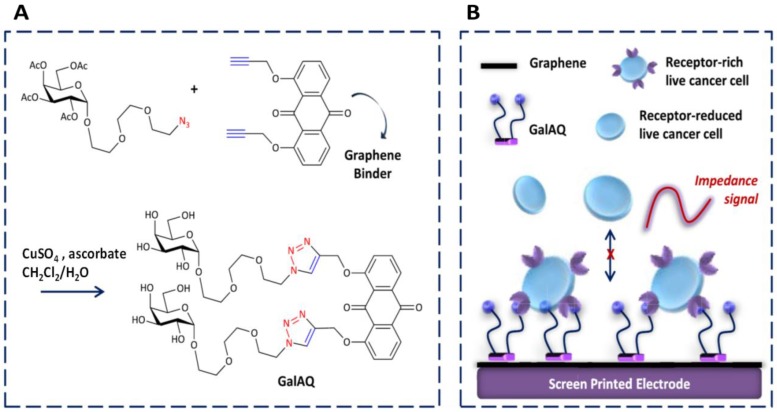
Synthesis of Gal-AQ (**A**) and scheme of the label-free impedance detection of receptor-rich live cancer cells (**B**). Reproduced from [66] with permission.

The detection of cells with conventional biochemical techniques usually requires the lysis of cells to release a biomarker, with risk of deterioration as well as long times and difficult manipulation, making simpler and more direct methods desirable. In this context, Cui et al. [66] reported a method involving CuAAC for the synthesis of the recognition element. The strategy consisted of the construction of a galactosyl anthraquinone (Gal-AQ) dye for the label-free impedance detection of Hep G2 live cancer cells. A click Cu(I)-catalyzed reaction of an alkynyl anthraquinone with azido galactoside yielded the probe which was subsequently employed to recognize sugars after binding to a graphene-coated screen-printed electrode by self-assembly (Figure 13). Taking advantage of selective sugar-receptor recognitions, un-labeled live cancer cells captured by the electrode produced an increase in the charge transfer resistance (R_ct_) measured by EIS. Hep G2 cells were detected over a 2000–10,000 cells mL^−1^ linear range. Furthermore, to probe the ability of the biosensor to detect sugars, authors added increasing amounts of peanut agglutinin (PNA) to the Gal-AQ-modified electrode, observing a gradual increase in R_ct_ that suggested the binding of lectins to the glycosylic surface. However, insignificant impedance responses to a series of related lectins or proteins were observed.

Accessible folate receptors (FR) are normally expressed in cancer cells. FR is a glycosylphosphatidylinositol-anchored protein that binds folate with high affinity. Based on this strategy, electrochemical cytosensors involving CuAAC were developed [83]. Click chemistry was used to link propargyl-functionalized folic acid to an azide-modified Au paper working electrode (PWE) and azide-modified trimetallic dendritic nanoparticles (Au@PdPt NPs) to respectively capture and detect K-562 cells by attachment to their folate receptors in a sandwich-type configuration. Profiting the intrinsic peroxidase-like catalytic activity of Au@PdPt NPs for the reduction of H_2_O_2_ in the presence of THI, the electrochemical signals provided after the linking of the dendritic Au@PtPd NP nanoprobe to the cells were dramatically amplified. The biosensor exhibited a wide-detection linear range from 100 to 2.0 × 10^7^ cells mL^−1^ and an LOD of 31 cells mL^−1^ [67].

## 4. General Considerations, Challenges and Prospects

CuAAC provides a simple, rapid, versatile and attractive route to impart surfaces and nanomaterials with improved features in terms of selectivity and electrocatalytic properties that allow the incorporation of nanomaterials and/or biological compounds in a selective and controlled manner. So far, this chemistry has been used mostly to modify nanomaterials and conventional and disposable electrodes for preparing interfaces with enhanced operational and analytical characteristics for reliable electrochemical sensing and the enzymatic and affinity biosensing of relevant analytes using quite simple protocols.

In electrochemical sensing, this chemistry possesses a great versatility to: modify electrodes using electrografting in connection with electroactive or non-electroactive SAMs; functionalize carbon nanomaterials, mainly GO and CNTs, with β-CDs, ILs, PtNPs and BODIPYs; confer improved selectivity for electrodes modified with MPcs; and introduce functional groups in polymer networks. In addition, it has been shown that CuAAC is also an effective and attractive surface chemistry in the preparation of electrochemical biosensors enhancing the direct ET of immobilized enzymes and allowing effective surface modification, bioconjugation and signal transformation or amplification. Therefore, the great versatility and possibilities offered by CuAAC have been smartly exploited to develop DET enzymatic-, DNA-, immune- and peptide-based biosensors that exhibit improved operational and analytical behavior for the electrochemical biosensing of a wide variety of relevant target analytes for the resolution of major problems in food, environmental and clinical fields. However, despite the great progress demonstrated so far in exploiting to the fullest the unique features of this surface chemistry and making it advantageous (compared to much more advanced techniques such as the use of alkanethiols), additional efforts should be made for the individual modification of multielectrode surfaces, developing greener protocols, preparing multicomponent layers and improving the storage stability of the as-prepared sensing interfaces.

## Figures and Tables

**Table 1:** Electrochemical platforms prepared by Cu(I)-catalyzed azide–alkyne cycloaddition (click chemistry).

Electrode Substrate	“Clicked” Materials	Click Strategy	Electrochemical Platform	Application/Analyte	Transduction Technique	Analytical Characteristics	Ref.
AuE	azide-undecanethiol SAM-4-pentynoic further covalent binding of Hb	ascorbate + CuSO_4_ 24 h	AuE/Hb	O_2_/H_2_O_2_	amperometry	LR: 1.0–75 μmol L^−1^	[14]
BDDE	5-Oxo-5-(prop-2-ynyloxy) pentanoic acid-BDDE and azido-propyl-mannose or lactose	ascorbate + CuSO_4_ 24 h	carbohydrate-BDDE	lectin	EIS	LR: up to 0.1 μmol L^−1^ LOD: 5 nmol L^−1^	[15]
AuE	1-azidoundecan-11-thiol and propargyl-functionalized Fc	electroclick, CuSO_4_ 30 min; −0.65 V vs. Ag/AgCl	propargyl-Fc/AuE	Cu^2+^/yoghurt	DPV	LR: 10^−14^–10^−9^ mol L^−1^	[16]
AuE	1-azidoundecan-11-thiol-AuE and propargyl acrylate. MIP preparation by polymerization with MAAM and AIBN. HQ as the template	ascorbate + CuSO_4_ 24 h	MIP-AuE	HQ	amperometry	LR: up to 15 μmol L^−1^ LOD: 1.21 μmol L^−1^	[17]
AuE	1-azidoundecan-11-thiol-AuE and propargyl-ferrocene	AA + CuSO_4_, 10 h	propargyl-Fc-AuE	AA	EIS	LR: 5–10^3^ *pmol* L^−1^ LOD: 2.6 *p*mol L^−1^	[18]
AuE	4-ethylnylphenyl (4-EP) diazonium and azido-HRP	ascorbate + CuSO_4_ 24 h	HRP/AuE	H_2_O_2_	amperometry	5–930 μmol L^−1^	[19]
GCE	4-azidobenzenediazonium and ethynylpyridine	ascorbate + CuSO_4_ 3 h	FePc/GCE	hydrazine	amperometry	LR: 10–340 μmol L^−1^ LOD: 10 μmol L^−1^	[20]
GCE	alkynyl-cobalt phthalocyanine (TA-Co Pc) and polymerized 4-azido-polyaniline (PANI-N3)	CuBr, 3h	TA-CoPc-N3-PANI/GCE	eserine	SWV	LR: 0.156–2.45 μmol L^−1^ LOD: 0.175 μmol L^−1^	[10]
PtE	azido-thiophene and ethynylferrocene	CuBr, DMF, PMDETA, 48 h	PtE	H_2_PO_4_^−^ HP_2_O_7_^3−^	CV		[21]
GCE	4-azido-aniline diazonium-GCE and 10-undecyn-1-thiol-AuNPs	ascorbate + CuSO_4_ overnight	AuNPs-GCE	NO_2_^−^ oxidation	CV/amperometry		[22]
ITO	polymerized azido-EDOT with PSS and ethynyl-ferrocene	ascorbate + CuSO_4_ 24 h	PSS-PEDOT-ITO	DA	LSV	LR: 0.01–0.9 mmol L^−1^ LOD: 1 μmol L^−1^	[23]
GCE	4-azidoaniline and 4-ethynylpyridine	electroclick, CuSO_4_ CV (−0.6–0.9 V, n = 50)	FeTCPc/GCE	hydrazine	amperometry	LR: 0.2–1 mmol L^−1^ LOD: 6.4 μmol L^−1^	[24]
GCE	azide-AuNPs and alkyne-2-cyano-prop-2-yl-dithio-benzoate. MIP preparation by polymeri-zation with EGDMA, AIBN and PEG. Fenitrothion as template	ascorbate + CuSO_4_ 24 h	MIP-GCE	fenitrothion/cabbage, apple peel	DPV	LR: 0.01–5 μmol L^−1^ LOD: 8 nmol L^−1^	[25]
GCE	azide-MWNTs (N3-MWNTs) and 1-propargyl-3-butylimidazolium bromide (IL). MIP prepara-tion by polymerization with 4-vinylpyridine, EGDMA and AIBN. Tartrazine as template	ascorbate + CuSO_4_ 24 h	MIP-MWCNTs-IL@PtNPs/GCE	tartrazine	DPV	LR: 0.03–5.0 and 5.0–20 μmol L^−1^ LOD: 8 nmol L^−1^	[26]
BDDE	4-azido-aniline-BDDE and ethynyl-ferrocene or alkyne-modified ss-DNA	AA + CuSO_4_ (Fc); TBTA + CuBr (DNA), 12 h	Fc-BDDE or DNA-BDDE	electrode modification			[27]
GCE	4-azidobenzenediazonium and Fe(II) tetrakis (5-hexyn-oxy) phtalocyanine (FcPc)	Cu(PPh_3_)_3_Br; TEA 18 h	FePc-GCE	hydrazine	CV/amperometry	LR: 0.1–1.0 mmol L^−1^ LOD: 1.09 mmol L^−1^	[28]
GCE	4-azido-aniline diazonium-GCE and 10-undecyn-1-thiol-AuNPs	electroclick, CuSO_4_, 1 h −0.12 V vs. Ag/AgCl	AuNPs-GCE	electrode modification			[29]
SPCE	azide-PEG4-AuNP-PAMAM and acetylene-PEG4-SPCE	ascorbate, Cu(II),	azide-PEG4-NHS	Cu^2+^/water	DPSV after AuNPs dissolution	LR: 50–10^7^ *p*mol L^−1^ LOD: 2.8 *p*mol L^−1^	[30]
GCE	azide CdSe/ZnS QDs and Fe(II) tetra-kis (5-hexyn-oxy) phtalocyanine (FcPc)	ascorbate; CuSO_4_ 48 h	FePc-QDs-GCE	paraquat	DPV	LOD: 5.9 nmol L^−1^	[31]
GCE	4-azidobenzenediazonium and Mn(II) tetrahexynyl-phtalocyanine	Cu(PPh_3_)_3_Br; TEA 18 h	MnPc-GCE	hydrazine	amperometry	LR: 0.2–1.0 mmol L^−1^ LOD: 15.4 *p*mol L^−1^	[32]
AuE	azide-Cu-calix[6]azacryptand and alkyne-terminated thiol SAM	electroclick, Cu(6-Br TMPA) −0.30 V vs. SCE	Cu-calix[6] azacryptand-AuE	alkylamines	CV		[33]
AuE	4-azidobenzenediazonium and Co(II)-or Mn(II)-tetra-(4-propargyloxy) phen-oxy phthalocyanines (MTPrOPhOPcs)	CuI, DMF/ACN 3 h	MTPrOPhOPcs/AuE	H_2_O_2_	amperometry	LR: 10–80 μmol L^−1^ LOD: 12.5 μmol L^−1^ (Co); 4.9 μmol L^−1^ (Mn)	[34]
GCE	4-azidobenzenediazonium and Co(II) tetrakis 4-((4-ethynylbenzyl) oxy) phthalocyanine	Cu(PPh_3_)_3_Br; TMA 18 h	CoPc-GCE	hydrazine	amperometry	LR: 0.1–1.0 mmol L^−1^ LOD: 10.2 μmol L^−1^	[35]
ITO	alkynyl-manganese phtalocyanine (TA-MnPc) and 4-azido polyaniline (PANI-N3)	electroclick, CuSO_4_, 15 min; −0.15 V vs. Ag/AgCl	TA-MnPc-N3-PANI/ITO	fenitrothion	SWV	LR: 0.05–2.81 μmol L^−1^ LOD: 0.015 μmol L^−1^	[36]
GCE	tetrakis (5-hexyn-oxy) phthalocyanine (CoPc) and azido-aniline	Cu(PPh_3_)_3_Br 24 h	CoPc/GCE	Hg(II), Pb(II), Cu(II), Cd(II)	DPASV	LR: up to 0.1 mmol L^−1^ LOD: 82 (Hg); 328(Cu); 56(Pb); 347(Cd) nmol L^−1^	[37]
GCE	azide-SWCNTs and BODIPY	ascorbate + CuSO_4_ 24 h	BODIPY-SWCNTs/GCE	guanine (G) adenine (A)	DPV	LOD: 1.07 μmol L^−1^ (G); 2.91 μmol L^−1^ (A)	[38]
GCE	azide-SWCNTs and BODIPY	ascorbate + CuSO_4_ 24 h	BODIPY-SWCNTs/GCE	eserine/orange juices	SWV	LR: 0.25–2.25 μmol L^−1^ LOD: 160 nmol L^−1^	[39]
GCE	azide CdSe/ZnS QDs and Fe(II), Co(II) or Mn(II) tetrakis 4-((4-ethyl-benzyl) oxy) phtalocyanine (MPc)	Cu(PPh_3_)_3_Br; TMA 72 h	MPc-QDs-GCE	H_2_O_2_	amperometry	LR: 0.1–1.0 mmol L^−1^ LOD: 0.023 μmol L^−1^ (Co)	[40]

AA: ascorbic acid; AIBN: azobisisobutyronitrile; BODIPY, 4,4-difluoro-8-(4-hydroxyphenyl)-2,6-diethynly-1,3,5,7-tetramethyl-4-bora-3a,4a-diaza-s-indacene; (CuPPh_3_)_3_Br: bromotris (triphenylphosphine) copper(I): DA: dopamine; DBCO-NH_2_: dibenzocyclooctyne-amine; DTPA: dithiol phosphoramidite; EGDMA: ethylene glycol dimethacrylate; 6-eTMPA: 6-ethynyl-tris(2-pyridylmethyl) amine; FeTCPc: Fe(II)tetracarboxyphtalocyanine; HAS: human serum albumin; MAAM: *N*,*N*-methylene-bis (acrylamide); PAMAM, poly(amidoamine); EDOT: polyethylenedioxythiophene; PMDETA: *N*,*N*,*N*′, *N*″,*N*″-pentamethyldiethylenetriamine; PSS: poly(styrene-4-sulfonate); SPCE: screen-printed carbon electrode; TBTA: tris (benzyltri-azolyl-methyl) amine; TEA, triethylamine; TMPA: tris (2-pyridylmethyl) amine).

**Table 2:** Electrochemical biosensors prepared by Cu(I)-catalyzed azide–alkyne cycloaddition.

Electrode Substrate	Clicked Materials	Click Strategy	Electrochemical Biosensor	Application/Analyte	Transduction Technique	Analytical Characteristics	Ref.
AuE	1,4-dialkynylbenzene and azido-HRP	sodium ascorbate, CuSO_4_, 24 h	HRP-AuE	H_2_O_2_	amperometry	LR: 5–700 μmol L^−1^ LOD: 2.5 μmol L^−1^	[53]
SPCE	grafted 4-((trimethysilyl) ethynyl) diazonium and azido-HRP	electroclick, CuSO_4_, −0.2 V vs. Ag, few min	HRP-SPCE	H_2_O_2_	amperometry	LR: 5–50 mmol L^−1^ LOD: 0.5 mmol L^−1^	[54]
GCE	alkyne-IgG and azide-SWCNTs	AA, CuSO_4_, overnight	HRP-anti-IgG-IgG-SWCNT/GCE	IgG	amperometry (HQ/H_2_O_2_)	LOD: 30 *p*g Ml^−1^	[55]
Au-PWE	1-azidoundecan-11-thiol and alkyne-Ab1; azide-Fe_3_O_4_@SiO_2_ (MSN) and propargyl Ab2 and HRP	AA, CuSO_4_, 18 h	Ab2/HRP-MSN	micro-cystin-LR	DPV	LR: 0.01–200 μg mL^−1^ LOD: 4 ng mL^−1^	[56]
SPCE	azido-aptamer and ethynyl-modified SPCE	electroclick; CuSO_4_ −200 mV vs. Ag; 5 min	H-Eth-Ar-p-NO_2_-Ar-SPCE	OTA/beer	EIS Fe(CN)_6_^3−/4−^	LR: 1.25–500 ng L^−1^ LOD: 0.25 ng L^−1^	[57]
GCE	azido-aniline and alkyne-hIgG	electroclick; Cu (II) −0.4 V vs. Ag/AgCl, 30 min	HRP-anti-gIgG/anti-hIgG-hIgG-GCE	hIgG	amperometry (HQ/H_2_O_2_)	LR: 0.1–10 ng mL^−1^ LOD: 0.01 ng mL^−1^	[58]
AuE	bis(DTPA)-hexynyl and azide-PEG3-Biotin	electroclick, TBTA + CuSO_4_, −0.3 V vs. Pt, 3 h	Strep-Biotin-AuE	Biotin-HSA	EIS	LR: 10–10^4^ *p*g mL^−1^ LOD: 10 *p*g mL^−1^	[59]
SPCE	propargylamine-GO and azide-MSN; 4-pentinoic acid-Ab2 and azide-MSN	AA, CuSO_4_, 24 h	GO-MSN-Ab2-CA253-Ab1-GO/SPCE	CA 153	DPV	LR: 10^−3^–200 U mL^−1^ LOD: 2.8 × 10^−4^ U mL^−1^	[60]
AuNPs/SPCE	azido-UDT and alkynyl-LBA	AA, CuSO_4_ DMSO/Tris buffer, 30 min	[Ru(NH_3_)_6_]^3+^/Lys-LBA-UDT-AuNPs/SPCE	lysozime	SWV	LR: 1.0–50.0 *p*g mL^−1^ LOD: 0.3 *p*g mL^−1^	[61]
AuE	azido-PEDOT and acetylene-DNA	CuI, TBTA, DIPEA/DMSO; 20–24 h	sDNA-PEDOT/AuE	HCV	DPV	LR: 1–20 nmol L^−1^ LOD: 0.13 nmol L^−1^	[62]
GCE	azide-Jug and alkynyl-APAP-hapten	ascorbate, CuSO_4_H_2_O/t-butanol, 36 h	anti-APAP-APAP/poly (Jug-co-Jug-APAP)-GCE	APAP	SWV	DR: 1–50 nmol L^−1^ LOD: 10 *p*mol L^−1^	[63]
AuE	MB-hairpin 5′alkyne-ssDNA and 3′azide ssDNA	ascorbate + Cu (II)	Oligo-A/Oligo-B/hairpin/AuE	Cu^2+^	SWV	LR: 5.0–1000 nmol L^−1^ LOD: 1.23 nmol L^−1^	[64]
AuE	ssDNA hybridized with thiol-and azido-hairpin and ethynylferrocene	AA, CuSO_4_	hairpins/MCH/ssDNA-ethynylferrocene	ssDNA	DPV	LR: 1–1000 nmol L^−1^ LOD: 0.296 *p*mol L^−1^	[65]
SPCE	alkynyl anthraquinone (AQ) and azido galactoside (Gal)	ascorbate; CuSO_4_ H_2_O/CH_2_Cl_2_; overnight	GalAQ/Gr/SPCE	PNA, Hep-G2 cells	EIS Fe(CN)_6_^3−/4−^	LR: 100–900 nmol L^−1^ (PNA); 2000–10,000 cells mL^−1^	[66]
Au-PWE	propargyl-folic acid (FA) and: (a) azide-Au-PWE, (b) azide-Au@PtPdNPs	AA, CuSO_4_ DMF, 2h	Au@PtPdNPs-FA-cell-FA-Au-PWE	K562 cells	DPV (THI/H_2_O_2_)	DR: 100–10^7^ cells mL^−1^ LOD: 31 cells mL^−1^	[67]
PtCE	hydroxypropyl cellulose (HPC) and azide-Fc	CuBr, PMDETA, DMF, 24 h	HRP/HPC-Fc-PtCE	H_2_O_2_	amperometry	LR: 0.1–8 μmol L^−1^ LOD: 2.5 μmol L^−1^	[68]
GCE	alkyne-IgG and azide-MWCNTs	AA, CuSO_4_, overnight	poly-HRP-Strept-Biotin-antiTGF-TGFβ-anti-TGF-IgG-MWCNT/SPCE	TGF-β1	amperometry (HQ/H_2_O_2_)	LR: 5–200 *p*g mL^−1^ LOD: 1.3 *p*g mL^−1^	[69]
GCE	4-azido aniline and 4-pentynoyl -peptide	ascorbate, CuSO_4_ 18 h	Strept-Biotin-peptide/Jug-Ph-NH_2_-GCE	PSA	SWV	DR: 10^−12^–10^−6^ mol L^−1^	[70]
BDDE	TIPs-Eth-Ar and azide peptide[(Ala-Arg-Leu-Pro-Arg)_2_Lys-Lys(N3)] and alkyne-terminated-BDD	ascorbate, CuSO_4_ TBTA, methanol/H_2_O 24 h	IFV-peptide-BDDE	H1N1, H3N2 IFV	EIS Fe(CN)_6_^3−/4−^	DR: 400–8000 pfu mL^−1^	[71]
AuE	ssDNA hybridized with thiol- and azido-hairpin and ethynylferrocene	electroclick; CuSO_4_ CV-0.2-0.3 V vs. SCE	hairpins/MCH/ssDNA-ethynylferrocene	ssDNA	DPV	LR: 1–1000 *p*mol L^−1^ LOD: 0.62 *p*mol L^−1^	[72]
AuE	alkyne-Fc-modified aptamer and azide UDT/UDT SAM	electroclick, Cu(II) −0.5 V vs. Ag/AgCl	Fc-aptamer-UDT/N3-UDT/AuE	VEGF165	amperometry	LR: up to 0.5 μmolL^−1^ LOD: 6.2 nmol L^−1^	[73]
AuE	azido-DNA and alkynyl-DNA/HRP-AuNPs	ascorbate; Cu(II) AuNPs	HRP/AuNPs-DNA-alkyne-azido-DNA/AuE	Cu^2+^/water	DPV, H_2_O_2_ addition	LR: 1.0 amol L^−1^–10 mmol L^−1^ LOD: 0.38 amol L^−1^	[74]
AuE	BPEI-Fc/CB[7]-N3-GO and 5′alkyne-Apt(S1)	electroclick; Cu(II) CV (0.5 to −0.3 V) 10 min, 12 h incubation	BPEI-Fc-CB[7]-N3-GO/S1/MCH/S2/AuE	VEGF165	SWV	DR:10 fg mL^−1^–1ng mL^−1^ LOD: 8 fg mL^−1^	[75]
AuE	azido-labeled aptamer and ethynyl -ferrocene	electroclick; CuSO_4_ CV-0.3 V to 0.45 V	ethynylferrocene-N3 Apt1-thrombin-Apt2/AuE	thrombin/serum	DPV	LR: 0.1–1000 nmol L^−1^ LOD < 84 *p*mol L^−1^	[76]
GCE	azide-dsDNA and PA	AA, released Cu(II), 30 min	Cu-PDA-Ab2-CA242-PA/Ab1-PEI-GO/GCE	CA 242	SWV	LR: 10^−4^–100 U mL^−1^ LOD: 20.74 μU mL^−1^	[77]
AuSPE	azide-terminated aptamers and propargylacetic acid	ascorbate, CuSO_4_ THPTA; 7 h	PEI/rGO/AuSPE	BNP cTnI	DPV Fe(CN)_6_^3−/4−^	LR:1–10^6^ (BNP); 1–10^3^ *p*g mL^−1^ (cTnI); LOD: 0.9 (BNP); 1 *p*g mL^−1^ (cTnI)	[78]
GCE	alkyne-IgG and azide-MWCNTs	AA, CuSO_4_, overnight	poly-HRP-Strept-Biotin-anti CXCL7-CXCL7-anti-CXCL7-IgG-MWCNT/SPCE	CXCL7/serum	amperometry (HQ/H_2_O_2_)	LR: 0.5–600 *p*g mL^−1^ LOD: 0.1 *p*g mL^−1^	[79]

APAP: acetaminophen; BPEI: branched: poly(ethyleneimine); CA 242: carbohydrate antigen 24-2; CB[7]: cucurbit[7]uril; DR: dynamic range; Fc: ferrocene; HCV: hepatitis C virus; IFV: influenza virus; Jug-Ph-NH_2_ Redox transducer 2-[(4-aminophenyl)sulfanyl]-8-hydro-xy-1,4-naphthoquinone; LBA: antilysozyme binding aptamer; LR: linear range; Lys: lysozyme; MCH: 6-mercapto-1-hexanol; MSN: magnetic silica nanoparticles; OTA: ochratoxine A; PA: propiolic acid; PEI: poly(ethyleneimine); PNA: peanut agglutinin; PtCE: platinized carbon electrodes; PWE: paper working electrode; TBTA: tris(benzyltriazolylmethyl)amine; THPTA: tris(3-hydroxypropyl triazolylmethyl) amine; THI, thionine; UDT, undecan-1-thiol.
